# Transmission of Severe Fever with Thrombocytopenia Syndrome Virus by *Haemaphysalis longicornis* Ticks, China

**DOI:** 10.3201/eid2405.151435

**Published:** 2018-05

**Authors:** Lu Zhuang, Yi Sun, Xiao-Ming Cui, Fang Tang, Jian-Gong Hu, Li-Yuan Wang, Ning Cui, Zhen-Dong Yang, Dou-Dou Huang, Xiao-Ai Zhang, Wei Liu, Wu-Chun Cao

**Affiliations:** Affiliated Bayi Children’s Hospital, PLA Army General Hospital, Beijing, China (L. Zhuang);; Beijing Key Laboratory of Pediatric Organ Failure, Beijing (L. Zhuang);; Beijing Institute of Microbiology and Epidemiology, Beijing (L. Zhuang, Y. Sun, X.-M. Cui, J.-G. Hu, L.-Y. Wang, D.-D. Huang, X.-A. Zhang, W. Liu, W.-C. Cao);; Center for Disease Control and Prevention of Chinese People’s Armed Police Forces, Beijing (F. Tang);; The 154 Hospital, People’s Liberation Army, Xinyang, China (N. Cui, Z.-D. Yang)

**Keywords:** severe fever with thrombocytopenia syndrome, severe fever with thrombocytopenia syndrome virus, SFTSV, Haemaphysalis longicornis, transovarial transmission, transstadial transmission, microinjection, ticks, China, viruses, vectorborne infections

## Abstract

We demonstrate maintenance and transmission of severe fever with thrombocytopenia syndrome virus by *Haemaphysalis longicornis* ticks in the larva, nymph, and adult stages with dissemination in salivary gland, midgut, and ovarian tissues. The *H. longicornis* tick is a competent vector to transmit this virus in both transovarial and transstadial modes.

Severe fever with thrombocytopenia syndrome (SFTS) is an emerging infectious disease caused by SFTS virus (SFTSV), identified in China in 2009 (*1*) and subsequently in South Korea (*2*) and Japan (*3*). Symptoms of SFTS usually include fever, thrombocytopenia, and leukocytopenia; case-fatality rates are 10%–30% (*1*,*4*). SFTS is implicated as largely a tick-associated disease, supported by evidence that many patients had exposure to ticks before disease onset (*1*). The longhorned tick, *Haemaphysalis longicornis,* the most abundant human-biting tick species in most SFTS-endemic areas of China (*5*), was found to harbor SFTSV (*1*,*6*,*7*). These studies suggested that *H. longicornis* ticks might be competent vectors for SFTSV transmission. Our study was designed to determine the role of the *H. longicornis* tick as a vector in maintenance and transmission of SFTSV.

## The Study

**Figure 1:**
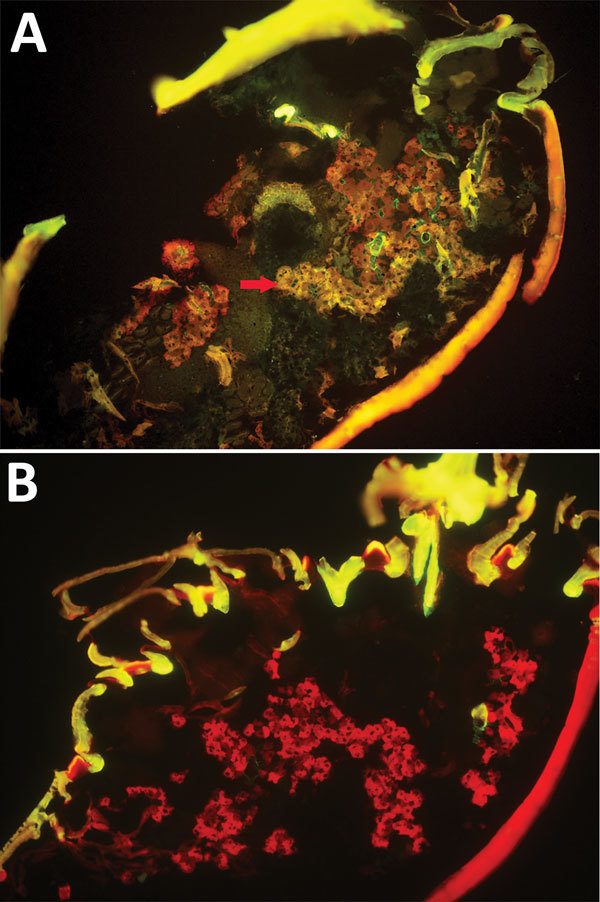
Specific detection of severe fever with thrombocytopenia syndrome virus (SFTSV) in microinjected *Haemaphysalis longicornis* ticks by indirect fluorescence assay. A) SFTSV-injected ticks 12 days after microinjection (original magnification ×10). Red arrows indicate specific fluorescence s. B) Phosphate-buffered saline–injected ticks 12 days after microinjection (original magnification ×10). No specific fluorescene is seen.

We randomly allocated 90 female *H. longicornis* ticks from an SFTSV-free colony into 2 equal groups, experimental and control. We injected the experimental group with SFTSV and the control group with phosphate-buffered saline (PBS). Seven days postinjection, we used 18 of the 35 live SFTSV-infected ticks for the detection of viral RNA by real-time reverse transcription PCR (rRT-PCR) (Technical Appendix); all showed positive results, confirmed by subsequent rRT-PCR and sequencing analysis. Twelve days postinjection, we dissected 5 live ticks from the experimental group to detect SFTSV in salivary glands and ovaries by indirect fluorescence assay (IFA), which showed notable SFTSV-specific fluorescence (Figure 1, panel A). For the control group, none of the 19 ticks tested by rRT-PCR had SFTSV RNA, and none of the 5 ticks tested by IFA showed SFTSV-specific fluorescence (Figure 1, panel B). 

We then let the remaining 12 live ticks from both groups feed on naive Balb/C mice (4 ticks/mouse) until the ticks detached from the mice. The engorged females were harvested and maintained to lay eggs. We determined transovarial transmission of SFTSV by further testing of SFTSV RNA from eggs, larvae, and nymphs using rRT-PCR. A total of 15 pools of eggs laid by 5 infected *H. longicornis* ticks (3 pools from each tick, each pool coming from a single female) were SFTSV RNA positive. In contrast, the egg pools from ticks of the control group were all negative. When hatched to larvae, 20 of 25 pools derived from the infected *H. longicornis* ticks (5 pools from each tick) tested positive for SFTSV RNA; all 25 larvae pools of the control group tested negative (Table 1).

**Table 1:** Detection of severe fever with thrombocytopenia syndrome virus RNA in experimental and control *Haemaphysalis longicornis* ticks

Source	Experimental infection group		Control group

No. tested	% Positive ± SE	No. tested	% Positive
Mother tick carcasses	5	100		5	0
Egg pool*	15	100		15	0
Larvae pool†	25	80.0 ± 1.7		25	0
Engorged larvae pool‡	25	100		25	0
Nymph pool§	25	92.0 ± 1.4		25	0
Engorged nymph	25	100		25	0
Male adult	25	36.0 ± 5.4		25	0
Female adult	25	44.0 ± 2.9		25	0
Female hemolymph pool¶	3	66.7 ± 9.4		4	0
Female saliva pool#	4	75.0 ± 14.4		4	0
Male hemolymph pool¶	3	33.3 ± 9.4		4	0
*Eggs were tested in pools of 60. †Larvae were tested in pools of 50. ‡Engorged larvae were tested in pools of 5. §Nymphs were tested in pools of 5. ¶Hemolymph collected from 5 ticks was pooled as 1 sample. #Saliva collected from 5 ticks was pooled as 1 sample.					

We further performed transstadial transmission of SFTSV by rearing larvae to adults. All remaining larvae were reared to nymphs and adults by feeding on 20 naive Balb/C mice. We subjected 1 mouse to SFTSV RNA testing after it was bitten by each pool of larvae and the hatched nymphs and adults; we used the other mice for feeding multiple pools of larvae and the hatched nymphs and adults. We fed 3,195 larvae in the experimental group and 2,987 in the control group to engorgement and randomly selected and tested engorged larvae. We maintained the other engorged larvae for molting to nymphs. In all, 694 engorged larvae in the experimental group and 652 engorged larvae in the control group successfully molted to nymphs (Technical Appendix Tables 1, 2).The remaining 569 nymphs in the SFTSV group and 527 nymphs in the control group were fed on 20 naive Balb/C mice; 453 nymphs in the SFTSV group and 437 in the control group were fully engorged. We divided the remaining engorged nymphs into 5 replicate cohorts; 166 engorged nymphs (39% ± 6% standard error [SE]) in the experimental group and 155 (38% ± 5% SE) in the control group matured to adults (Technical Appendix Tables 1, 2). The overall hatching rate of eggs and molting rates of nymphs and adults in the 2 groups were comparable.

All 25 engorged larvae pools (5 pools from each mother) in the SFTSV-infected group and none from the control group were positive for SFTSV RNA (Table 1). After the larvae molted to nymphs, 23 of 25 nymph pools from the SFTSV-infected group and none from the control group tested positive for SFTSV RNA. Similarly, all 25 engorged nymph pools from the SFTSV-infected group and none from the control group were positive. When the second generation emerged, we tested 50 adults (25 females, 25 males) in each group for SFTSV RNA; in the SFTSV-infected group, 44% (11/25) of the females and 36% (9/25) of the males tested positive, whereas all 25 females and 25 males in the control group were negative. Positive samples were confirmed by identical sequences to that of the inoculated virus strain.

A total of 83 naive Balb/C mice were infested by ticks (Technical Appendix Table 3). All 3 Balb/C mice fed by the SFTSV-infected females were positive for exposure to SFTSV 1 week after the ticks detached. Of the naive Balb/C mice that were bitten by larvae from the SFTSV-infected group, 4 of 5 were positive for SFTSV RNA, as were 4 of 5 mice bitten by nymphs, 4 of 5 mice bitten by adult female ticks, and 3 of 5 mice bitten by male ticks; mice bitten by ticks from the control group were negative (Table 2). We used IFA to test serum samples from the mice collected before and 3 weeks after detachment of ticks at different developing stages; all mice positive for SFTSV RNA demonstrated seroconversion against SFTSV (Table 2).

**Table 2:** Detection of severe fever with thrombocytopenia syndrome virus in *Haemaphysalis longicornis* tick–infested mice*

Stage (sex)	No. mice	No. ticks/mouse	No. positive by rRT-PCR		No. positive by IFA	Titer ± SE
Experimental group	Control group
Adults (female)	3	4	3	0	3	3.01 ± 0.30
Larvae	5	50	4	0	4	2.78 ± 0.15
Nymphs	5	10	4	0	4	3.16 ± 0.17
Adults (female)	5	5	4	0	4	3.09 ± 0.15
Adults (male)	5	5	3	0	3	2.81 ± 0.35

*IFA, indirect fluorescence assay; rRT-PCR, real-time reverse transcription PCR.

**Figure 2:**
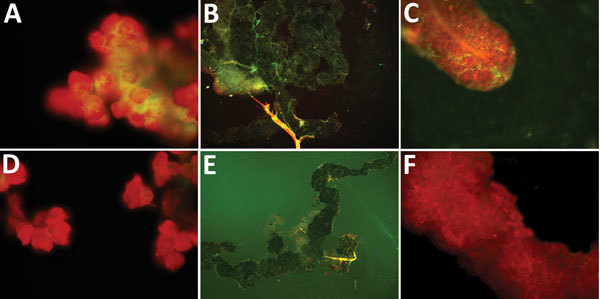
Specific detection of severe fever with thrombocytopenia syndrome virus (SFTSV) in tissues of adult *Haemaphysalis longicornis* ticks by indirect fluorescence assay. The green fluorescence represents the SFTS virus.  A) Salivary gland of SFTSV-injected tick (original magnification ×40). B) Midgut of SFTSV-injected *H. longicornis* tick (original magnification ×10). C) Ovary of SFTSV-injected tick (original magnification ×40). D) Salivary gland of phosphate-buffered saline (PBS)–injected tick (original magnification ×40). E) Midgut of PBS-injected *H. longicornis* tick (original magnification ×10). F) Ovary of PBS-injected tick (original magnification ×40).

Three of 4 pools of saliva and hemolymph from the experimental group were SFTSV RNA positive. We selected 5 females at random from each group to detect SFTSV in tissues by IFA. The salivary glands, midguts, and ovaries of the SFTSV-injected group displayed SFTSV-specific fluorescence (Figure 2).

We observed a significantly higher level of viral load in second-generation eggs than in second-generation adults (p<0.001 by Mann-Whitney U-test). We also found a significantly higher level (p<0.0001) of viral load in saliva of engorged second-generation adults than in saliva of unengorged adults, indicating that SFTSV had multiplied.

## Conclusions

We report the experimental maintenance and transmission of SFTSV in *H. longicornis* ticks. After microinjection of SFTSV, the virus disseminated in ovaries and salivary glands. Infected *H. longicornis* ticks could transmit SFTSV successfully in both transovarial and transstadial modes. The appearance of SFTSV in saliva and hemolymph suggests that the virus circulates in the tick hemocoel and is expressed in saliva. In addition, naive Balb/C mice infested with experimentally infected adults, larvae, and nymphs all became infected, evidenced by both detection of SFTSV-specific RNA and seroconversion. 

These findings, together with data on natural infection in the field (*1*,*6*), implicate *H. longicornis* ticks as competent vectors for SFTSV. However, the evidence derived from IFA and rRT-PCR tests could not indicate that the virus is infectious. More efforts should be taken to demonstrate the infectivity of SFTSV in the transmission cycle. 

*H. longicornis* ticks are widely distributed in the Asia–Pacific region (*8*–*12*). Predominant hosts of *H. longicornis* ticks include humans, poultry, livestock, wild rodents, and birds (*12*–*14*). As displayed in mice in the current research, SFTSV is likely to be maintained through vertical and horizontal transmission in ticks that infest these wild and domestic mammals. This maintenance has been evidenced by an extraordinarily high prevalence of SFTSV in sheep, cattle, dogs, pigs, and other animals (*7*,*14*). In areas where *H. longicornis* ticks are endemic, infested animals could be considered as key reservoirs in maintaining and transmitting SFTSV (*15*). The close contact between animals and their owners could pose another way of acquiring infection, in addition to tick bites.

Technical Appendix Materials and methods for detection of severe fever with thrombocytopenia syndrome in *Haemaphysalis longicornis* ticks.
