# Supplementary material for: Transmission of Severe Fever with Thrombocytopenia Syndrome Virus by Haemaphysalis longicornis Ticks, China
Source: Emerg Infect Dis. 2018 May;24(5):868–71. doi: 10.3201/eid2405.151435 (PMC5938789; doi:10.3201/eid2405.151435)
Supplement: Technical Appendix — Materials and methods for detection of severe fever with thrombocytopenia syndrome in Haemaphysalis longicornis ticks. [file 15-1435-Techapp-s1.pdf]

Article DOI: https://doi.org/10.3201/eid2405.151435

Transmission of Severe Fever with Thrombocytopenia Syndrome Virus by *Haemaphysalis longicornis* Ticks

Materials and Methods

SFTSV Strain and Culturing

The severe fever with thrombocytopenia syndrome virus (SFTSV) (Phlebovirus WCH/97/HN/China/2011, GenBank accession nos. JQ341190, JQ341189, and JQ341188, for L, M, and S segments) used in this study was isolated from a patient in Henan Province of China in 2011 (*1*), and maintained in the Vero E6 cell line with complete Dulbecco's modified Eagle's medium (DMEM), 10% fetal serum, and 10 U/mL penicillin and streptomycin. After we determined viral loads by quantitative real-time PCR, we harvested the virus for artificial infection of ticks by microinjection.

Tick Colony and Rearing

H. longicornis ticks were collected by flagging on vegetation in Shangcheng County, Henan Province,China in 2011. We established SFTSV-free tick colonies in our laboratory from engorged females. Briefly, the *H. longicornis* ticks were allowed to feed on Balb/C mice. All mice in this study were 2-week-old males, specific-pathogen free, supplied by the Center of Experimental Animals, Academy of Military Medical Sciences, China. The fully engorged females were kept individually until they laid eggs. We randomly sampled 10 batches (30 eggs in each batch) of eggs to screen for SFTSV, along with the corresponding adult mother tick, by isolation and RT-PCR assays, described later. The eggs from the groups in which both the mother tick and the filial eggs were negative for SFTSV were incubated to larvae. The larvae and

the following nymphs were fed on Balb/C mice, and the molted adults were subjected to the trial. The transmission cycle of SFTSV in *H. longicornis* ticks was simulated following the procedures shown in Technical Appendix Figure 1, with each step described as follows.

Artificial Infection of Ticks with SFTSV by Microinjection

Adult female *H. longicornis* ticks from the aforementioned SFTSV-free colony were infected with SFTSV by the microinjection protocol developed by Kocan et al. (*2*) with modification. We injected 1 μL of virus culture (5.9×10^5 copies/mL) into each tick through its anal pore with a microsyringe (1 inch, 33 gauge needle) under a dissecting stereomicroscope (Technical Appendix Figure 2). We injected the same volume of phosphate-buffered saline (PBS) into ticks that were used as the control group. The ticks that were crawling and active after injection were maintained in an Intelligent Climate Cabinet (Saife Company, Ningbo City, China) with a relative humidity of 95 ± 5% at 22°C.

Transmission Cycle of SFTSV in Ticks

Two weeks after injection, the female ticks were fed on Balb/C mice so we could investigate transovarial transmission. The engorged female ticks were maintained until they laid eggs, which were allowed to hatch to larvae under the same conditions as described earlier. We screened subsequent larvae for SFTSV infection to assess the efficiency of transovarial transmission. Larvae and subsequent nymphs were allowed to feed on Balb/C mice until fully engorged and molt to nymphs and adults. At each developmental stage, ticks were starved for 3 weeks between molting and the next feeding. We tested the derived nymphs and adults for SFTSV to evaluate the efficiency of transstadial transmission.

Detection of SFTSV in Ticks of Different Developmental Stages

We subjected ticks of different developmental stages to real-time PCR and RT-PCR to determine their SFTSV infection status. We extracted RNA from egg pools (60/pool), larva pools (50/pool), nymph pools (5/pool) (Technical Appendix Table 2), and individual adult ticks

using the RNeasy Mini Kit (QIAGEN, Hilden, Germany) following the manufacturer's instructions. For real-time PCR assay, we used the one-step Primer Script RT-PCR Kit (TaKaRa, Dalian, China) according to the manufacturer's instructions with 1 μLPCR primer mix (20 μM of sense and antisense each), 0.5 μL probe (10 μM) and 2 μL total RNA in LightCycler 2.0 (Roche Diagnostics, Mannheim, Germany). The real-time PCR primers (5′-ACCTCTTTGACCCTGAGTTWGACA –3′ and 5′- CTGAAGGA GACAGGTGGAGATGA-3′) and probe (5′-Hex-TGCCTTGACGATCTT-MGB-3) were targeted at the S-segment of the SFTSV (*3*). We performed RT-PCR and sequencing of the S-segment on positive samples (BNYS1-F: 5′-TCTTCTCCATCAAGAACAGC-3′, BNYS1-R: 5′-TTCGACAAAATTAGACCTCC-3′) to verify the real-time PCR results.

We prepared the positive control standard (nt. 1456–1557 of the SFTS virus segment S sequence, reference sequence GenBank accession no. KC505134) as described previously (*4*). We prepared serial dilutions from 10^8 to 10^3 copies/mL in diethylpyrocarbonate-treated water and stored them in RNase-free tubes at –80°C.

We performed quantification of SFTSV as described earlier in the 7500 real-time PCR system (Applied Biosystems, Foster City, CA, USA). Absolute RNA quantification was included in every assay and was generated by using RNA transcripts produced earlier. Standard curves included 5 dilutions and 3 replicate wells for each dilution. All samples were quantified in 3 replicate wells. Levels of SFTSV RNA concentrations were expressed as copies/mL.

Detection of SFTSV in Saliva and Hemolymph of Adult Ticks

To prepare the molted adult ticks to salivate, we allowed them to engorge partially by feeding on Balb/C mice. Saliva was collected (Technical Appendix Figure 3, panel A) from the engorged ticks as described previously (*5*). After saliva collection, we obtained hemolymph samples from each tick by clipping a front leg and placing the tip of a glass micropipette to the wound (Technical Appendix Figure 3, panel B). The saliva from 5 ticks in each group was

pooled and the hemolymph from the same 5 ticks was pooled, mixed with 140 μL PBS, and subjected to the RNA extraction procedure using the QIAamp Viral RNA Mini Kit (QIAGEN). We then applied the RNA to RT-PCR assay, for which positive amplicons were sequenced as mentioned earlier.

IFA Detection of SFTSV in Ticks

Twelve days following injection with SFTSV, we embedded the whole bodies of the ticks in paraffin and cut them longitudinally at a cryostat (Leica CM 3050; Leica Microsystems, Wetzlar, Germany). We put the frozen slices on glass slides and subjected them to immunofluorescent assay (IFA) for SFTSV detection.

We then selected 10 females at random from the SFTSV and control groups and individually dissected their guts, salivary glands, and ovaries under sterile conditions using a dissecting microscope. After marginal cuts and scutum removal with lancets, we carefully removed the hemolymph around the tissues with filter papers and replaced it with sterilized PBS 3 times. We placed the tick tissues on glass slides and subjected them to IFA for SFTSV detection.

We soaked sheet glasses with prepared tissues in PBS with 5% skim milk to deparaffinize. We incubated the slices at 37°C for 1 hour with mAb that was previously prepared (*6*) in PBS with 0.05% Tween20. We used serum from Balb/C mice asa negative control. Following triple washing with PBS and 1 final washing with distilled water, we incubated the slices at 37°C for 30 minutes with fluorescence-conjugated goat antimouse antibodies (Zhongshanjinqiao, Beijing, China) at 1:100 dilution with Evans blue. We washed the slices in PBS 3 times and finally washed them with distilled water, visualizing with an Olympus BX51 Microscope until dried.

Detection of SFTSV in Mice

We collected serum samples from the mice 3 times (before tick feed, 1 week after tick engorgement, and 3 weeks after tick repletion) and extracted RNA using a QIAamp Viral RNA mini kit and detection by real-time PCR, as described earlier. By using the viral antigen of the SFTSV patient source from the Vero E6 cell line, we detected specific IgG against SFTSV by indirect IFA, as previously described. We measured antibody titers with serum dilution starting at 1:16 and then serially 2-fold to determine endpoint titers.

Statistical Analysis

The antibody reciprocal titers were log-transformed. We used the Mann-Whitney test to determine the difference of SFTSV viral load in generation 2 eggs and adults, as well as the difference between the viral load of unengorged generation 2 adults and saliva collected from engorged generation 2 adults.

References

1. Lam TT, Liu W, Bowden TA, Cui N, Zhuang L, Liu K, et al. Evolutionary and molecular analysis of the emergent severe fever with thrombocytopenia syndrome virus. Epidemics. 2013;5:1–10. PubMed http://dx.doi.org/10.1016/j.epidem.2012.09.002

2. Kocan KM, Blouin E, de la Fuente J. RNA interference in ticks. J Vis Exp. 2011; (47):2474. http://dx.doi.org/10.3791/2474

3. Zhang YZ, He YW, Dai YA, Xiong Y, Zheng H, Zhou DJ, et al. Hemorrhagic fever caused by a novel bunyavirus in China: pathogenesis and correlates of fatal outcome. Clin Infect Dis. 2012;54:527–33. http://dx.doi.org/10.1093/cid/cir804

4. Sun Y, Liang M, Qu J, Jin C, Zhang Q, Li J, et al. Early diagnosis of novel SFTS bunyavirus infection by quantitative real-time RT-PCR assay. J Clin Virol. 2012;53:48–53. http://dx.doi.org/10.1016/j.jcv.2011.09.031

5. Spielman A, Ribeiro JMC, Mather TN, Piesman J. Dissemination and salivary delivery of Lyme disease spirochetes in vector ticks (Acari: Ixodidae). J Med Entomol. 1987;24:201–5. http://dx.doi.org/10.1093/jmedent/24.2.201

6. Hofmann H, Li X, Zhang X, Liu W, Kühl A, Kaup F, et al. Severe fever with thrombocytopenia virus glycoproteins are targeted by neutralizing antibodies and can use DC-SIGN as a receptor for pH-dependent entry into human and animal cell lines. J Virol. 2013;87:4384–94. http://dx.doi.org/10.1128/JVI.02628-12

Technical Appendix Table 1. Numbers (± standard error) of generation 2 ticks used in each stage of the transmission cycle

Source	SFTSV group	Control group
Eggs	1305.6 ± 47.7	1436.6 ± 159.9
Eggs for detection (eggs × pools)	60×3	60×3
Eggs left	1125.6 ± 47.7	1256.6 ± 159.9
Hatched larvae	889 ± 66.2	847.4 ± 114.2
Larvae for detection (larvae × pools)	50×5	50×5
Larvae left	639 ± 66.2	597.4 ± 114.2
Engorged larvae	185.2 ± 24.5	179.4 ± 5.6
Engorged larvae for detection (larvae × pools)	5×5	5×5
Engorged larvae left	160.2 ± 24.5	154.4 ± 5.6
Nymphs	138.8 ± 19.7	130.4 ± 10.6
Nymphs for detection (nymphs × pools)	5×5	5×5
Nymphs left	113.8 ± 19.7	105.4 ± 10.6
Engorged nymphs	90.6 ± 14.9	87.4 ± 10.7
Engorged nymphs for detection (nymphs × pools)	5×1	5×1
Engorged nymphs left	85.6 ± 14.9	82.4 ± 10.7
Adults	33.2 ± 6.1	31 ± 4.6
Female adults	24.2 ± 8.9	23.2 ± 5.3
Female adults for detection	5	5
Hemolymph (females × pools)	5×1	5×1
Saliva (females × pools)	5×1	5×1
Male adults	9 ± 4	7.8 ± 2.5
Male adults for detection	5×1	5×1
Hemolymph (males × pools)	5×1	5×1

Technical Appendix Table 2. Mean days in each period of development of *H. longicornis* ticks in the SFTSV study (± standard error)

	Days	
Period	SFTSV group	Control group
Preovipositon period	8.20 ± 2.39	7.8 ± 1.80
Oviposition period	7.73 ± 1.69	7.7 ± 1.72
Egg hatching period	38.50 ± 1.24	38.4 ± 1.46
Larva feeding period	3.52 ± 0.64	3.4 ± 0.63
Larva premolt period	14.50 ± 1.85	14.2 ± 2.37
Larva molting period	7.70 ± 1.67	7.9 ± 1.64
Nymph feeding period	5.80 ± 1.28	5.55 ± 1.20
Nymph premolt period	16.90 ± 2.79	16.75 ± 2.52
Nymph molt period	10.20 ± 1.35	9.95 ± 1.42
Adult feeding period	9.12 ± 1.42	9.04 ± 1.84

Technical Appendix Table 3. Numbers of mice for feeding in the life cycle of the *H. longicornis* ticks in the SFTSV study

	SFTSV group		Control group	
Source	Mice for feeding and detection (ticks/mouse)	Mice for feeding	Mice for feeding and detection (ticks/mouse)	Mice for feeding
Generation 1 adults	3 (4)	0	3 (4)	0
Eggs	0	0	0	0
Larvae	5 (50)	5	5 (50)	5
Nymphs	5 (10)	5	5 (10)	5
Generation 2 female adults	5 (5)	5	5 (5)	5
Generation 2 male adults	5 (5)	4	5 (5)	3

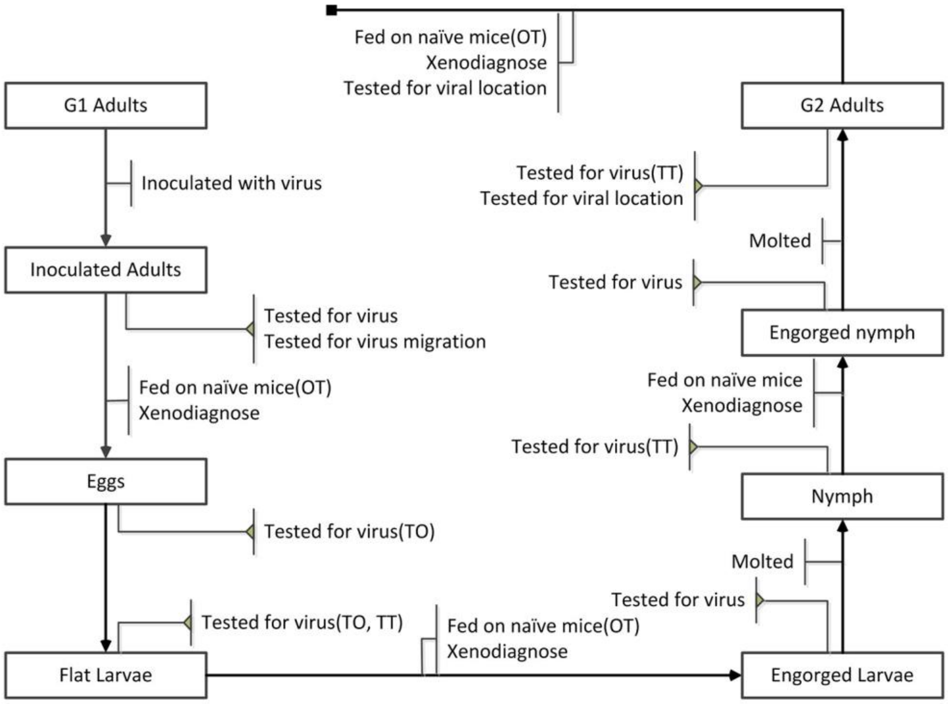

Technical Appendix Figure 1. Experimental framework of *H. longicornis* ticks microinjected with SFTSV. We microinjected 45 adult *H. longicornis* ticks with SFTSV cell culture dilution (5.92×10^5 copies/mL) and microinjected 45 other adult ticks with PBS for negative control. Ticks were then fed on Balb/c mice. All control ticks were found to be SFTSV negative. To evaluate the SFTSV transmission from ticks to mice, we performed reverse transcription PCR and IFA of mice serum samples. To evaluate transovarial and transstadial transmission, we detected SFTSV RNA by PCR of 15 pools of eggs, 25 pools of larvae, 25 pools of nymphs, 25 males, and 25 females. We collected 4 pools of saliva and blood lymph from molted adults (generation 2) and used reverse transcription PCR assay for detection. We performed IFA of tissue smears of molted adults (generation 2) to show the localization of SFTSV. OT, oral transmission; TO, transovarial transmission; TT, transstadial transmission.

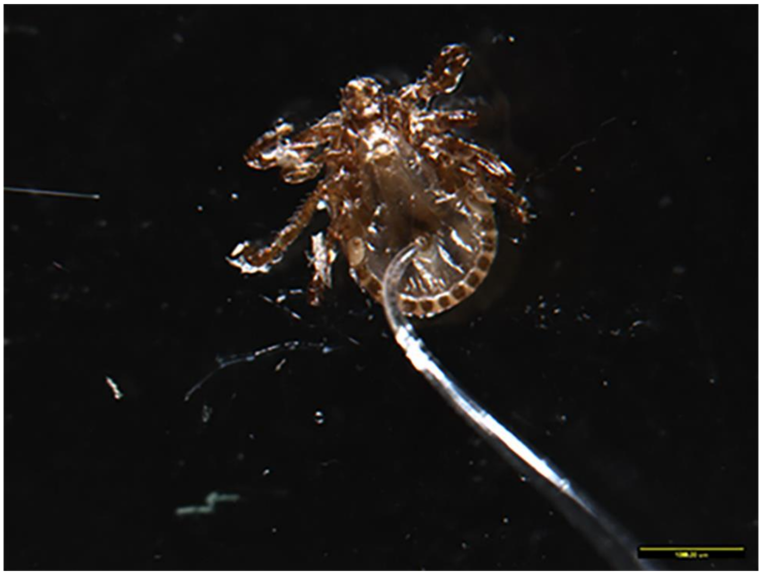

Technical Appendix Figure 2. Microinjection of adult *H. longicornis* ticks with SFTSV or PBS.

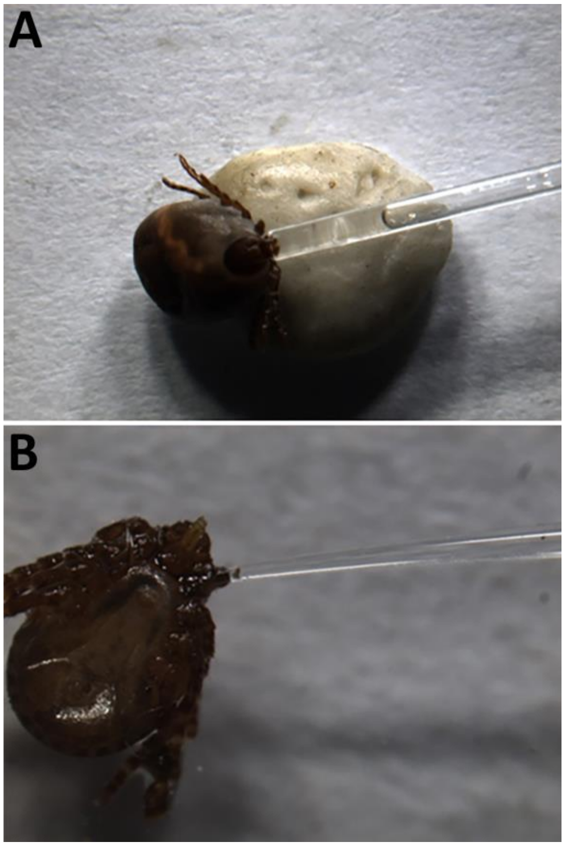

Technical Appendix Figure 3. Saliva and hemolymph collection from *H. longicornis* ticks. A) Collection of saliva from generation 2 adults. B) Collection of hemolymph from generation 2 adults.
